# Lower leg reconstruction after resection of a squamous cell carcinoma on necrobiosis lipoidica with a pedicled fibula and an extended anterolateral thigh flap—a case report

**DOI:** 10.1186/s12957-023-02923-z

**Published:** 2023-02-06

**Authors:** Olimpiu Bota, Friedegund Meier, Marlene Garzarolli, Klaus-Dieter Schaser, Adrian Dragu, Feras Taqatqeh, Hagen Fritzsche

**Affiliations:** 1 University Center for Orthopedics, Trauma and Plastic Surgery, Faculty of Medicine Carl Gustav Carus, TU Dresden, Fetscherstraße 74, 01307 Dresden, Germany; 2 Department of Dermatology, Faculty of Medicine Carl Gustav Carus, TU Dresden, Fetscherstraße 74, 01307 Dresden, Germany

**Keywords:** Extended cutaneous scquamous cell carcinoma, Necrobiosis lipoidica, Pedicled fibula transplantation, Anterolateral thigh perforator flap, Reconstructive microsurgery, Surgical oncology

## Abstract

**Background:**

Extensive loss of soft tissue and bone due to neoplasia, trauma, or infection in extremities often leads to amputation.

**Case presentation:**

We present the case of a 72-year-old female patient presenting with an extended cutaneous squamous cell carcinoma of the lower leg, developed on top of necrobiosis lipoidica. After achieving the R0 resection, a 26 × 20-cm soft tissue and 15-cm tibial bone defect resulted. The contralateral leg had been lost due to the same disease 18 years before. We achieved a successful reconstruction of the leg using a pedicled fibula transplantation, an extended anterolateral thigh perforator flap, and an internal fixation with plate and screws. Two years after the original surgery, the patient is relapse-free and mobile, with adequate function of the reconstructed foot.

**Conclusions:**

Our case presented a unique combination of pedicled fibula transplantation and free extended ALT perforator flap to reconstruct an extensive defect after resection of a rare cSCC on top of NL. In selected cases, the boundaries of limb salvage can be pushed far beyond the current standards of treatment.

**Supplementary Information:**

The online version contains supplementary material available at 10.1186/s12957-023-02923-z.

## Background

Cutaneous squamous cell carcinoma (cSCC) is the second most common cancer in humans. It can agressively infiltrate local tissues and has a reported recurrence rate of 2.7 to 4.6% and a rate of metastasis of 1.2 to 4%. The locally spread disease can be treated by surgery, radiotherapy, or combined treatment. In cases of advanced local disease, anti-PD1 therapy can be considered [1]. Necrobiosis lipoidica (NL) is a rare chronic idiopathic granulomatous disease that usually appears in the lower legs. The lesions have a tendency for ulceration and, in rare cases, a cSCC may develop on NL [2].

Large bony defects of the tibia can be reconstructed by the Masquelet technique, by bone transport, or by vascularized bone transplantation [3]. While the free vascularized fibula flap is a well-known technique to bridge large bone gaps, the pedicled fibula transplantation is also a feasible technique, without inducing supplementary donor site morbidity in the contralateral leg [4].

The free anterolateral thigh (ALT) flap has become over the years a workhorse flap in reconstructive surgery. With a reliable blood supply, it can be harvested as an extended flap to cover extremely large soft tissue defects [5].

## Case presentation

**Fig. 1:**
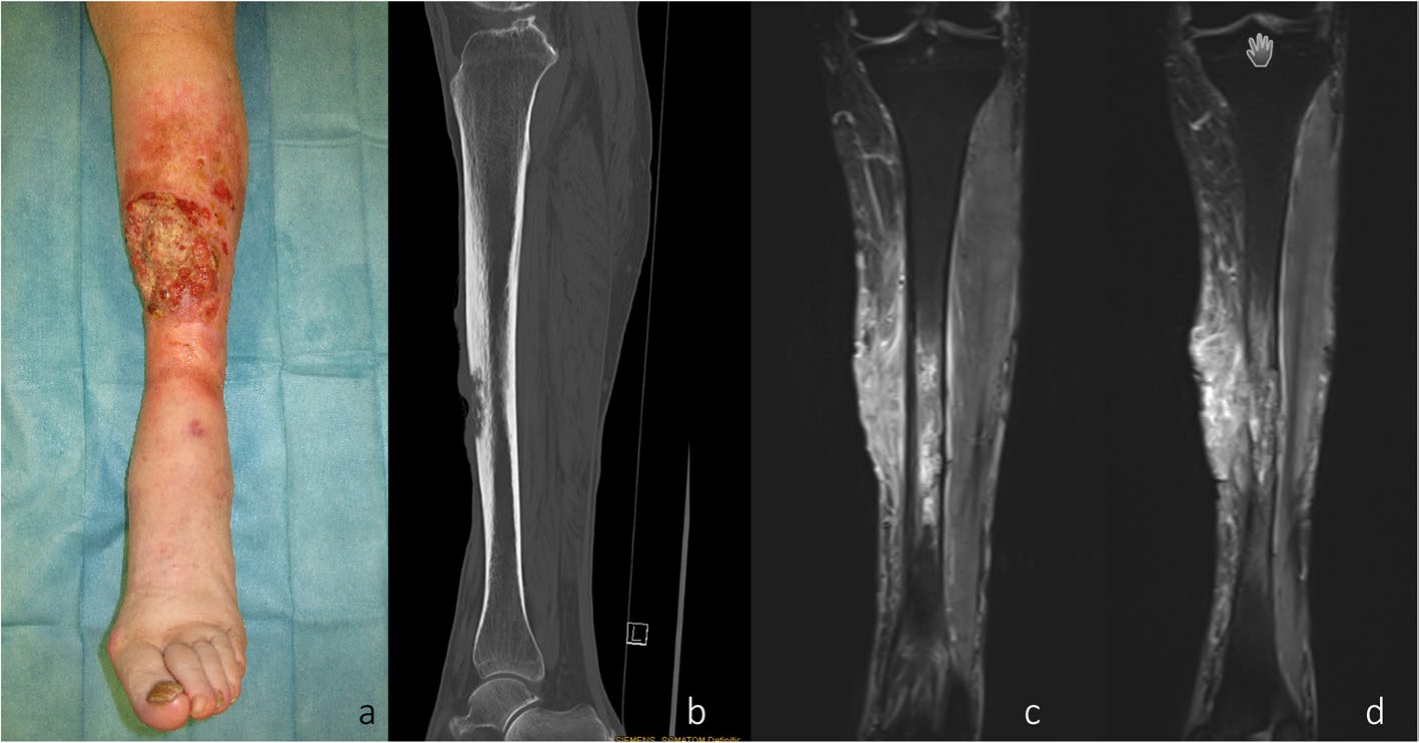
**a** Preoperative clinical photo. **b** Sagittal slice of preoperative CT scan (latero-lateral). **c** Coronal slice of preoperative MRI. **d** Coronal slice of preoperative MRI

**Fig. 2:**
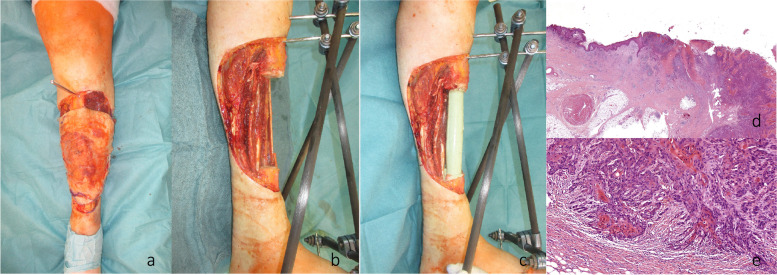
**a** Intraoperative photo of the leg before en bloc tumor resection. **b** Leg after en bloc resection and external fixation. **c** Leg after implantation of the PMMA spacer.** d** Histopathological images with cSCC, 3 × magnification.** e** Histopathological images with cSCC, 8 × magnification

**Fig. 3:**
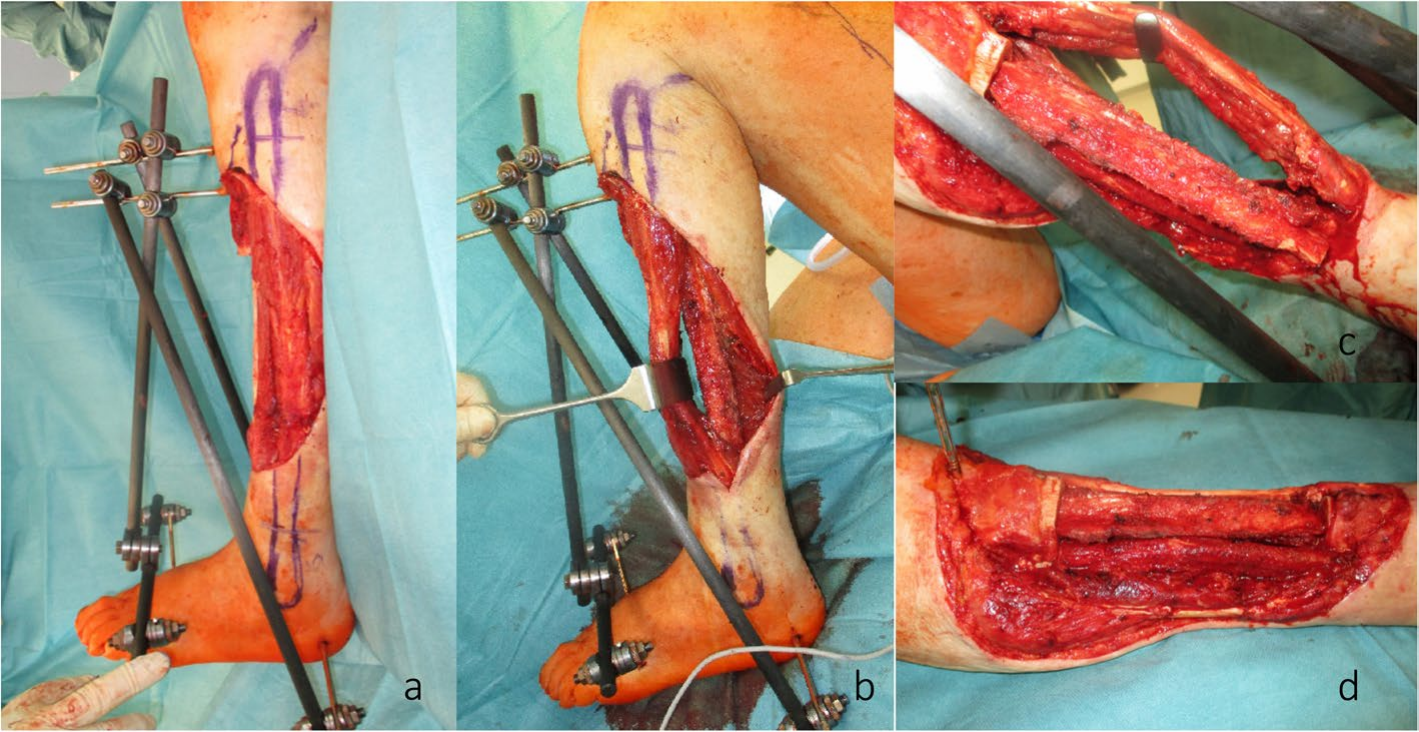
**a** Intraoperative planning before fibula harvest. **b** Pedicled fibula before transposition. **c** Transposed fibula as press fit into the tibial stumps. **d** Reconstructed tibia before plate osteosynthesis

**Fig. 4:**
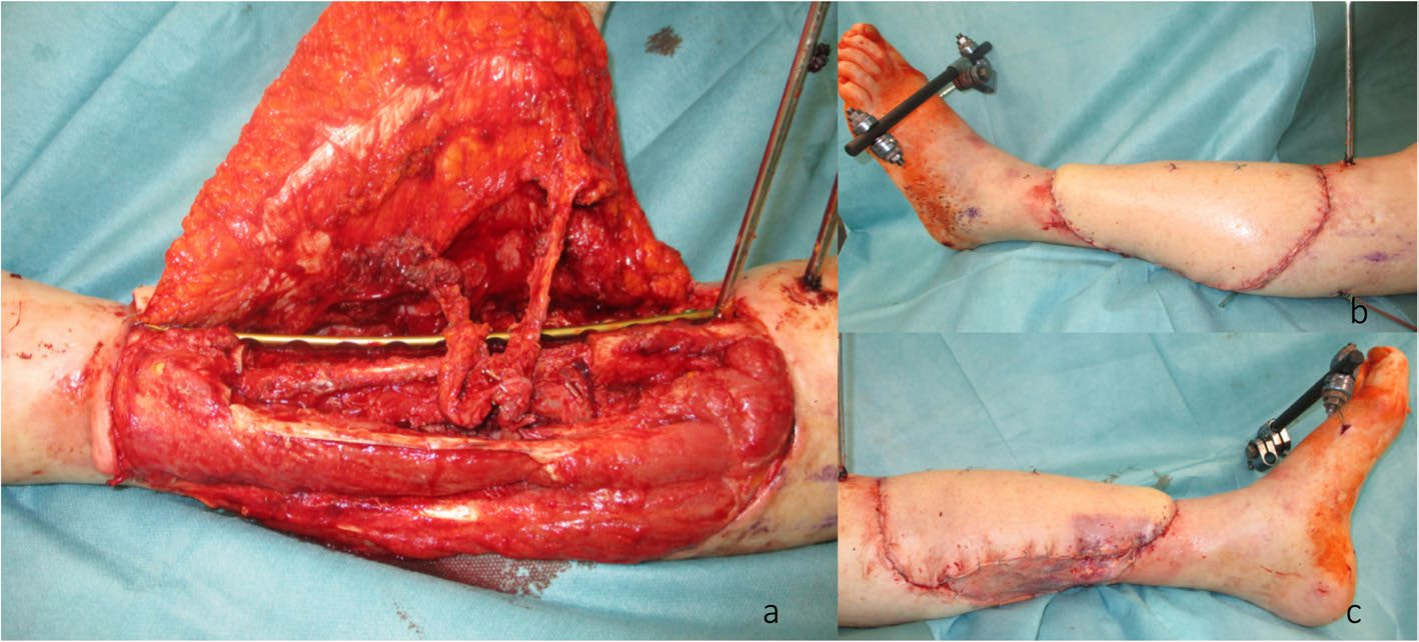
**a** Reconstructed tibia with the plate osteosynthesis and the extended ALT flap with the two perforator vessels.** b** Lateral aspect of the leg after reconstruction.** c** Medial aspect of the leg after reconstruction

**Fig. 5:**
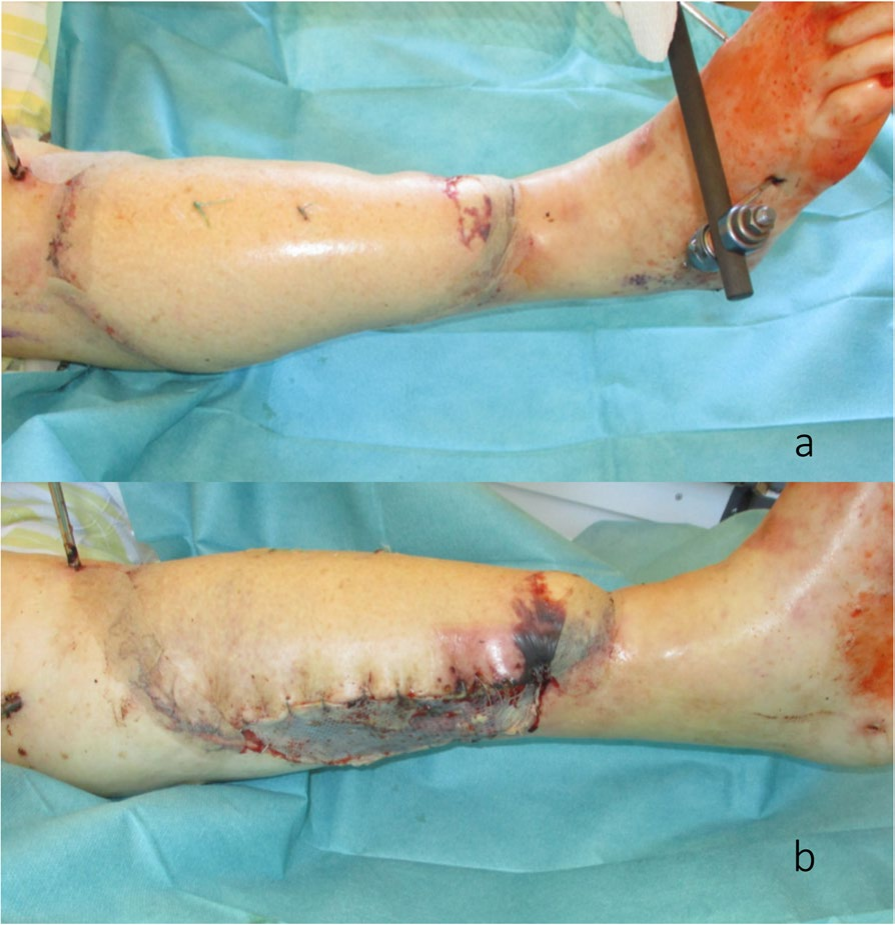
**a** Leg 4 days postoperative with reduced perfusion of the distal part, anterior view.** b** Leg 4 days postoperative with reduced perfusion of the distal part, medial view

**Fig. 6:**
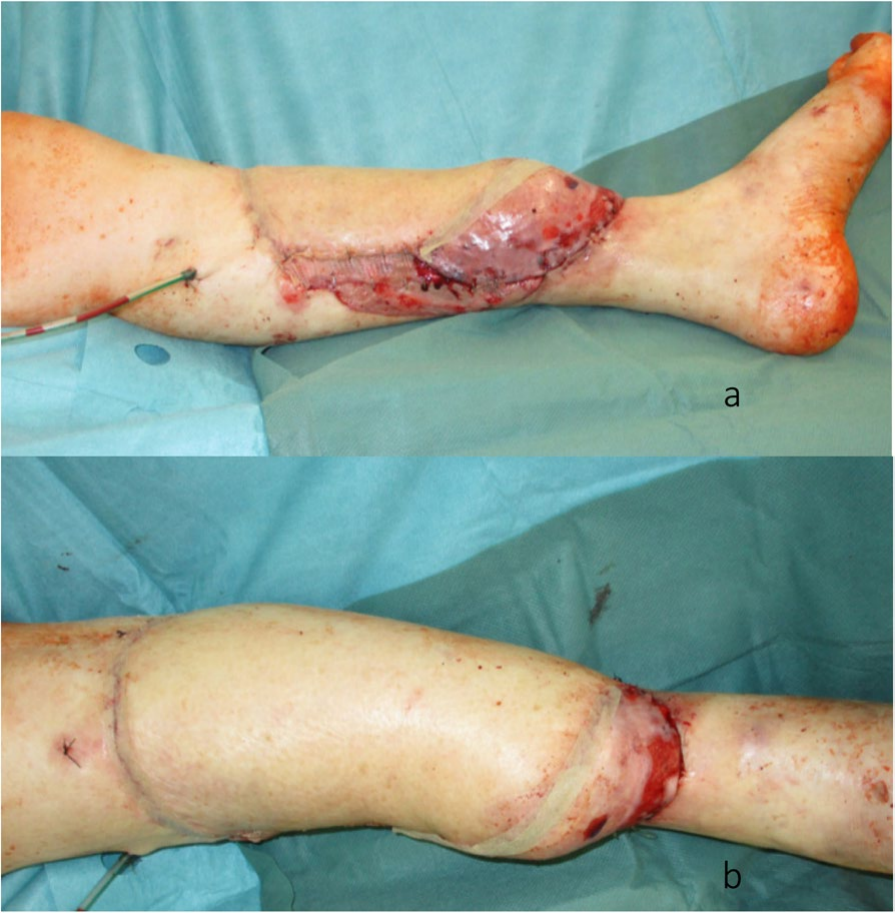
**a** Final aspect after reconstruction with a second ALT flap in the distal part of the leg, medial view; the red color with missing epidermis depicts the split thickness skin donor site.** b** Final aspect after reconstruction with a second ALT flap in the distal part of the leg, anterior view

We hereby present the case of a 72-year-old woman with an extended cSCC of the leg with tibial infiltration, developed on a chronic ulcer that developed on top of NL, without diabetes mellitus (Fig. 1a–d). Eighteen years before, the patient had developed the same condition on the contralateral leg and a below the knee amputation had been performed. After biopsy for confirmation and staging including whole body CT scan and regional lymph node sonography, the tumor board recommended the curative resection of the localized tumor. A curative en bloc resection of the tumor together with the soft tissue and 15 cm of involved tibia was performed (Fig. 2a,b). A polymethyl methacrylate (PMMA) spacer was used to bridge the tibia and an external fixator stabilized the leg (Fig. 2c). The wound was temporarily closed using negative wound pressure therapy (NPWT). The histopathological findings showed macroscopically a 165 × 152-mm large tumor, microscopically with 22-mm invasion of the fatty tissue and 10-mm invasion of the tibia, with a at least 18-mm tumor-free margin on the surface and at least 5 mm deep (pT4a, pNx, L0, V0, Pn0, G2, R0) (Fig. 2d,e). After achieving the R0 resection, a reconstruction plan was developed. Digital subtraction angiography was performed, showing a three-vessel supply of the leg. Being the only leg in an otherwise healthy patient who was mobile with a leg prosthesis on the right side, the indication for limb preservation was established. Seven days after the first surgery, the reconstructive surgery took place. The PMMA spacer was removed and a proximal pedicled fibular bone flap was harvested. The bone length was 18 cm. The bone ends were then beveled to fit the tibial medullary cavity and the fibula was then press-fitted in the tibia, reconstructing the 15-cm bone gap with 1.5 cm of fibula lying proximally and distally in the tibia (Fig. 3a–d). Two centimeters of proximal fibula was resected to ensure a tension-free pedicle positioning. A plate and screw osteosynthesis was performed, bridging the fibula graft, but fixating the beveled edges to the tibia. The external fixator was now removed. The muscles of the anterior and lateral compartment remained supplied by the anterior tibial vascular pedicle. The remaining soft tissue defect of 26 × 20 cm was measured and a template was transferred to the right thigh, centered on the descending branch of the lateral circumflex femoral artery. An extended ALT fasciocutaneous flap measuring 26 × 14 cm including two perforator vessels was harvested. The vessel anastomosis at the recipient site was performed to the anterior tibial artery as a flow-through flap and to the venae commitantes using vessel couplers (Fig. 4a). The medially exposed gastrocnemius muscle was split skin grafted (Fig. 4b,c). The donor site was also split skin grafted from the contralateral side. Postoperatively antibiotic treatment was initiated. The distal part of the flap (8 × 4 cm) showed a demarcation due to inadequate perfusion (Fig. 5a,b). At revision surgery, after flap debridement, pus emptied from the plate surroundings. A thorough debridement with lavage of the site was performed. NPWT was used for one cycle to control the infection. *Bacteroides fragilis* could be isolated and the antibiotics were adapted to the antibiogram. At the next surgery, further debridement and lavage with the exchange of the plate and screws was performed. The remaining soft tissue defect was covered with a second ALT flap measuring 16 × 7 cm from the left thigh, including the fresh split skin donor sites, using two perforator vessels (Fig. 6a,b). The flap anastomosis was performed end-to-side to the posterior tibial vessels, distal to the first anastomosis. The donor site was closed primarily. Postoperatively, all wounds showed primary healing. The antibiotics were administered for a total of 6 weeks starting from the last surgery. After 4 weeks, mobilization with partial weight-bearing of the leg was initiated. Twelve weeks postoperatively, ambulation using a wheeled walker was started.

One year postoperatively, the CT scan showed insufficient bony consolidation at the proximal and distal tibia-fibula transition despite exogenic ultrasound therapy, so a revisional surgery was performed. The extended ALT flap was longitudinally split to provide access to the plate while avoiding the lesion of the vascular pedicle. Two osteosynthesis plates were used to stabilize the leg medially and laterally and to ensure bone consolidation. Autologous cancellous bone was applied at the transition between the fibula and tibia proximally and distally. Postoperatively, the wounds healed without issues. After 6 weeks, mobilization of the leg was started using a fitted boot with pressure distribution to the tibial tuberosity and the foot. After 3 months postoperatively, the exogenic ultrasound therapy was continued for another 3 months. The pressure distribution was progressively switched from the tibial tuberosity to the foot.

**Fig. 7:**
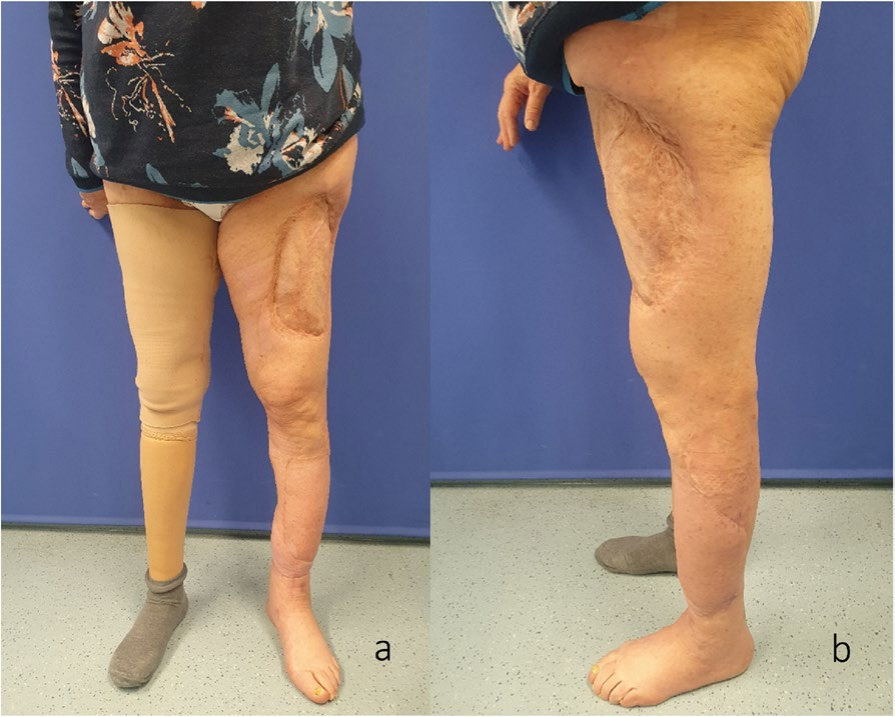
Patient standing 2 years after the first surgery; the left thigh depicts the extended ALT donor site, while on the right side, she wears a below-the-knee prosthesis. **a** Anterior view. **b** View from the left side

**Fig. 8:**
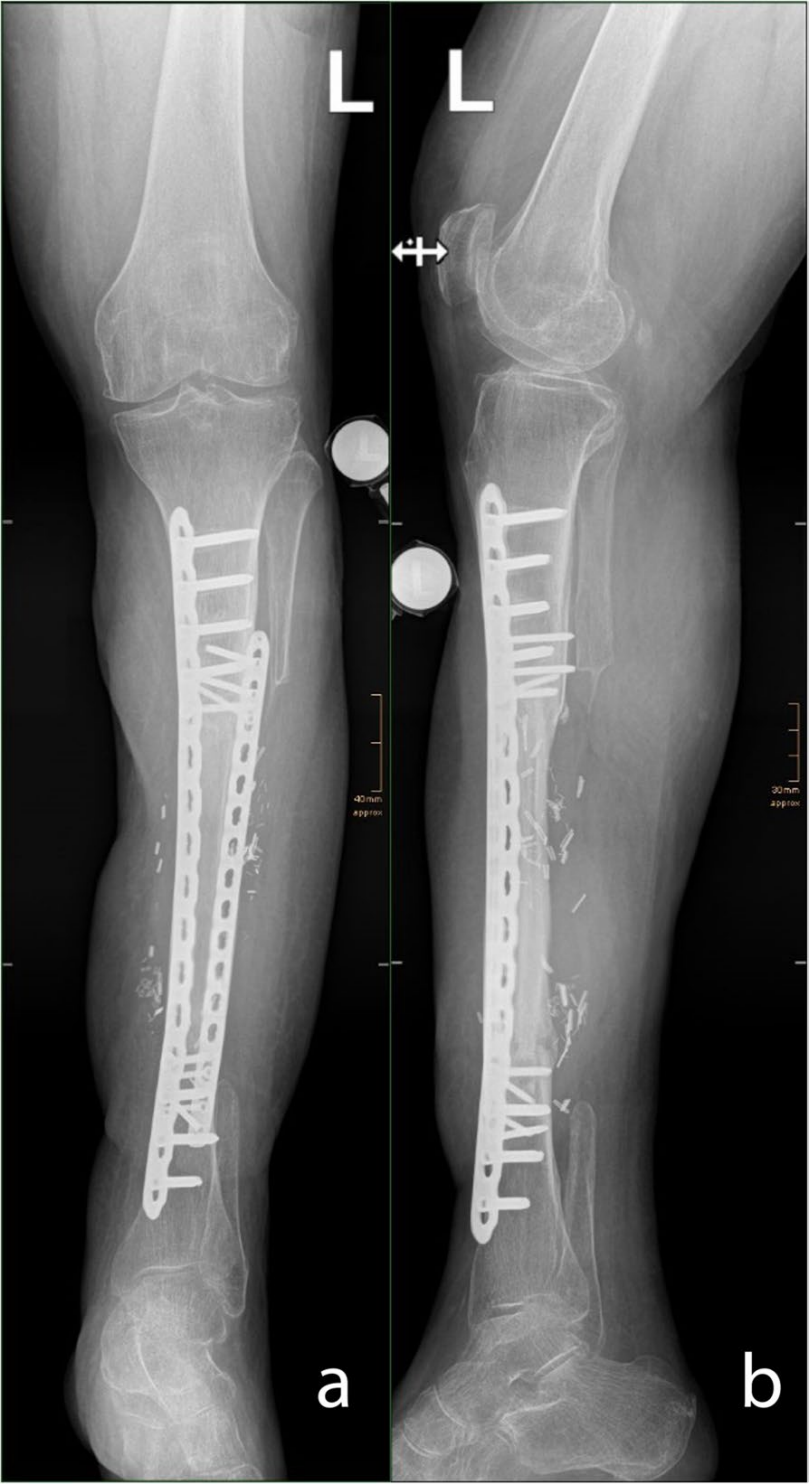
X-rays 2 years after the first surgery with the two plates and the fibular hypertrophy. **a** Anterior posterior view. **b** Lateral view

**Fig. 9:**
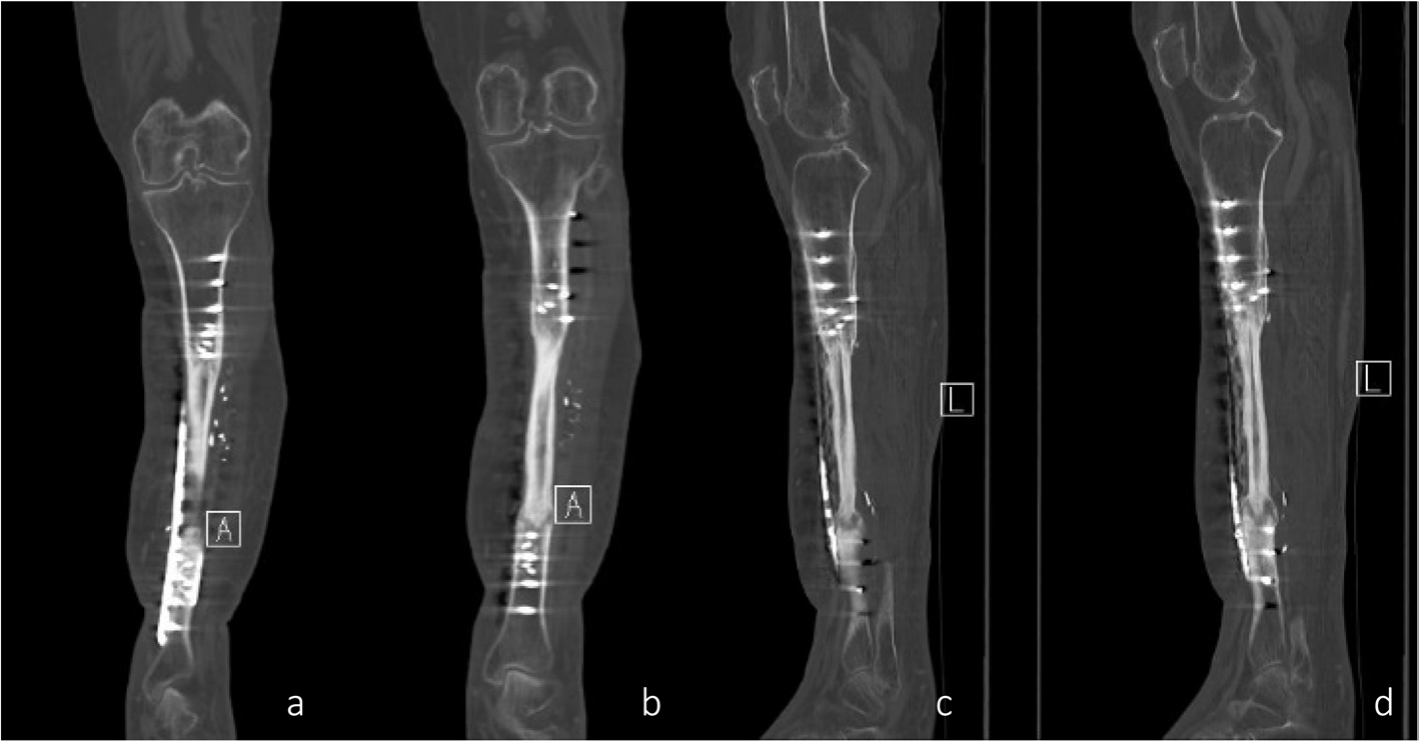
CT scan 2 years after the first surgery. **a,** **b** Coronal slices. **c, d** Sagittal slices

Two years after the beginning of treatment, the boot was completely removed. The patient was mobile on the reconstructed leg and below-the-knee prosthesis on the contralateral side (Fig. 7a,b). The extension of the foot was actively possible (Video 1). The X-rays (Fig. 8a,b) and CT-scan (Fig. 9a–d) showed a fibular hypertrophy of about 90% of the tibia as well as no local recurrence of the tumor and no metastasis. Considering the age of the patient, R0 resection of the lesion, no lymph node metastasis or other metastasis, no adjuvant therapy was performed.

## Discussion and conclusions

In this case report, we present the successful treatment of a very complex bone and soft tissue defect of the lower leg using innovative microsurgical reconstructive techniques.

NL is a rare granulomatous disease, which in very rare cases may degenerate into cSCC. A literature review by de Souza et al. identified only 16 published cSCCs on top of NL [2]. Although when first described, NL was associated with diabetes mellitus (DM), the current literature shows no direct correlation between the two diseases. Johnson et al. retrospectively reviewed 97 patients with NL, out of whom only 32% had DM. They found that histologically epidermal acanthosis was more commonly seen in diabetic patients, while naked granulomas were more frequently seen in patients without DM [6]. Our patient had no DM.

Even if surgery is the first-choice treatment for cSCC, immune checkpoint inhibitors gained a leading role in the treatment of locally advanced and metastatic cSCCs. In our case, surgical therapy was preferred because the tumor was still resectable and an anti-PD1 therapy failure could not be ruled out in a cSCC arising in NL [7].

After R0 resection, a 15-cm tibial defect resulted. This defect could have been reconstructed using bone transport or the induced membrane technique described by Masquelet. Although technically feasible, we preferred due to the age of the patient and the dimension of the bony defect an interposition with vascularized bone to avoid complications like bone exposure or bone resorption. The free vascularized transfer of the contralateral fibula is a well-known procedure and ensures a fast interposition of the tibial defect, with the price of supplemental donor site morbidity in the contralateral, usually healthy leg. In our case, this procedure was rendered impossible by the existing lower leg amputation. As the ipsilateral fibula was healthy and not affected by the tumor resection, this appeared to be the fastest and safest solution for the patient. The salvage of the remaining leg was of utter importance for our 72-year-old patient. With a three-vessel blood supply of the leg, the fibula could be skeletonized with the fibular artery and veins as pedicle. The blood supply of the remaining musculature from the anterior and lateral compartments was secured by the anterior tibial artery and veins. Khira et al. reported over 12 patients who received a reconstruction of the tibial shaft with a pedicled fibula transfer [4]. After a mean follow-up of 38 months, there was a bony union of both fibular ends in 9 patients, with a mean percentage of hypertrophy of 95%, which corresponds to our patient. Manfrini et al. used a tibial allograft combined with a vascularized fibular graft to reconstruct the tibia in 47 patients [8]. Out of this cohort, they compared the 22 patients who received a free contralateral fibular graft with the 25 patients who received an ipsilateral fibular transfer and found that the latter showed shorter operative times, fewer technical challenges, and no morbidity on the contralateral leg. In both studies, there was no supplementary soft tissue coverage necessary.

After the first flap coverage, the patient developed an infection at the plate site. This could be attributed to the contamination of the medullary cavity by the exulcerated tumor. Although antiseptic surgical methods were employed and antibiotics were administered postoperatively, the infection developed probably due to the biofilm around the plate by an anaerobic bacterium which was not covered by the antibiotic treatment. After mobilization, an incongruent weight loading appeared to develop with instability on the lateral side of the fibula, which led to repeated microfractures and delayed union. Considering the contamination of the operative site and the difficult consolidation, an Ilizarov external fixator might have been an appropriate alternative for osteosynthesis from the start, as in the study of Khira et al. [4]. Nevertheless, the encountered complications in our case could be redressed while the patient profited from the internal fixation.

The transplantation of the fibula to a weight-bearing position implies periosteal hypertrophy, to match the size of the tibia, which is about double in size. The progressive transfer of weight bearing from the tibial tuberosity to the foot using the fitted boot assisted the fibular hypertrophy so that after 2 years, the fibula reached almost the diameter of the tibia.

ALT perforator flap is one of the most reliable workhorse flaps in reconstructive surgery. Harvested as extended, the ALT can cover extremely large soft tissue defects. Saint-Cyr et al. reported over 12 ALT extended flaps with a mean surface of 365 cm^2^ without partial necrosis [9]. In our case, with a flap of 330 cm^2^, although we harvested two perforator flaps with the pedicle, marginal distal necrosis developed. This could be attributed to the insufficient perfusion of the distal part of the flap and the infection which developed around the osteosynthesis material. The resulting defect after revision could be swiftly closed with a normal-sized contralateral ALT perforator flap, despite the still healing split-thickness donor area. Except for the functional and aesthetical advantages, fasciocutaneous flaps develop a reliable collateral blood supply. In our case, we could split the extended flap longitudinally a year after transplantation, without any impairment of the blood supply.

In this case, we showed that in selected cases, the boundaries of limb salvage can be pushed far beyond the current standards of treatment. Our case presented a unique combination of pedicled fibula transplantation and free extended ALT perforator flap to reconstruct an extensive defect after resection of a rare cSCC on top of NL.

## Supplementary Information

**Additional file 1: Video 1.** The extension of the foot was actively possible

## Data Availability

Not applicable.

## Abbreviations

ALT: Anterolateral thigh
cSCC: Cutaneous scquamous cell carcinoma
DM: Diabetes mellitus
NL: Necrobiosis lipoidica
NPWT: Negative wound pressure therapy
PMMA: Polymethyl methacrylate
